# Severe Pemphigus Vulgaris Resistant to Conventional Therapies and with Hypersensitivity to Rituximab in a 12-Year-Old Child

**DOI:** 10.3390/children10060920

**Published:** 2023-05-24

**Authors:** Maria Beatrice De Felici Del Giudice, Carolina Calanca, Chiara Sassetti, Carlo Caffarelli, Claudio Feliciani, Susanna Esposito

**Affiliations:** 1 Dermatology Unit, Department of Medicine and Surgery, Azienda Ospedaliero-Universitaria of Parma, 43126 Parma, Italy; bea.defelici@gmail.com (M.B.D.F.D.G.); claudio.feliciani@unipr.it (C.F.); 2 Pediatric Clinic, Department of Medicine and Surgery, Azienda Ospedaliero-Universitaria of Parma, 43126 Parma, Italy; ccalanca.carolina@gmail.com (C.C.); chiaracsassetti@gmail.com (C.S.); carlo.caffarelli@unipr.it (C.C.)

**Keywords:** bullous disease, desensitization, pediatric dermatology, pemphigus vulgaris, rituximab

## Abstract

Pemphigus vulgaris (PV) is a rare, potentially lethal blistering disease typically occurring in adulthood and characterized by autoantibodies directed against mucocutaneous desmosomal proteins. Clinically, flaccid vesicles, bullae and erosions after breakage are the main clinical features. According to the literature, the incidence of PV is rare in the pediatric population, ranging from 1 to 4% of reported cases. We describe an interesting case of a 12-year-old boy with severe PV that was referred to our university hospital for a mucocutaneous disease resistant to anti-infective therapy. Following the appearance of bullous lesions on the skin, antibody screening for autoimmune diseases showed positivity for PV and corticosteroid therapy was started. In view of the numerous adverse effects, we decided to set up biological therapy with rituximab, which was interrupted due to the onset of an urticarial reaction. Further second-line therapies were therefore attempted, with only a partial response. For this reason, a desensitizing therapy with rituximab was decided, thus allowing a clear improvement in the clinical picture and quality of life of the patient. To the best of our knowledge, this is the first report of a child with severe PV resistant to conventional therapies and with an urticarial reaction to rituximab. This case highlights that despite PV being extremely rare in the pediatric population, this diagnosis should not be entirely discounted. In case of severe clinical manifestations, rituximab represents a valid option in children and desensitization tests should be recommended in the presence of hypersensitivity to this drug.

## 1. Introduction

Pemphigus vulgaris (PV) is a very rare chronic bullous disease (1:1,000,000) which typically occurs in patients aged between 45 and 65 years, and it is characterized by the formation of autoantibodies, mainly of the IgG class, directed against desmoglein-1 and desmoglein-3 (Dsg1 and Dsg3 protein) [1]. Clinically, it is represented by flaccid intraepithelial vesicles which, after breaking, result in erosions at the mucocutaneous level. The suspicion of PV is clinical, confirmed by histopathology and laboratory investigations. High doses of corticosteroids are the first-line therapy in PV, eventually followed by integration with immunosuppressive drugs. In particular, the anti-CD20 monoclonal antibody rituximab (R) is currently the first-line therapy in adulthood [2,3]. Other immunosuppressants such as azathioprine and/or mycophenolate mofetil and/or methotrexate can be considered to obtain a steroid-sparing effect [2,3]. If there is no response to first-line therapy, it is possible to opt for intravenous (IV) infusion of immunoglobulins and/or plasmapheresis [2,3].

The occurrence of PV is even rarer in the pediatric population, ranging from 1 to 4% of reported cases [4]. We describe an interesting case of a 12-year-old boy with severe PV that was referred to the University Hospital of Parma, Parma, Italy, for a mucocutaneous disease resistant to anti-infective therapy. Remarkably, the main finding seems to be therapeutic success after a desensitizing therapy with R because of a hypersensitivity reaction to this drug and a marked improvement in the patient’s quality of life thanks to a multidisciplinary approach.

## 2. Case Presentation

We report the case of a 12-year-old boy who was admitted to our Pediatric Emergency Unit for severe gingivostomatitis and conjunctivitis present for 15 days and worsening despite oral treatment with amoxicillin–clavulanic acid and acyclovir.

Vaccination calendar was sorted by age, and his personal and familial medical history was negative for dermatological and allergic diseases. The child appeared in good general clinical condition and hydration, was apyretic, had rosy skin and an absence of appreciable cutaneous lesions. He showed an intense non-secreting bilateral conjunctival hyperemia combined with multiple erosions and widespread de-epithelialization of the gingival mucosa, in particular at the vestibular level and hyperemic pharynx with aphthous lesions to the tonsillar pillars. He also reported pain during swallowing. A diagnosis of Steven–Johnson syndrome (SJS) was ruled out due to the clinical features of the manifestation, the patient’s generally good condition, and the fact that the time evolution in SJS is very rapid.

**Figure 1:**
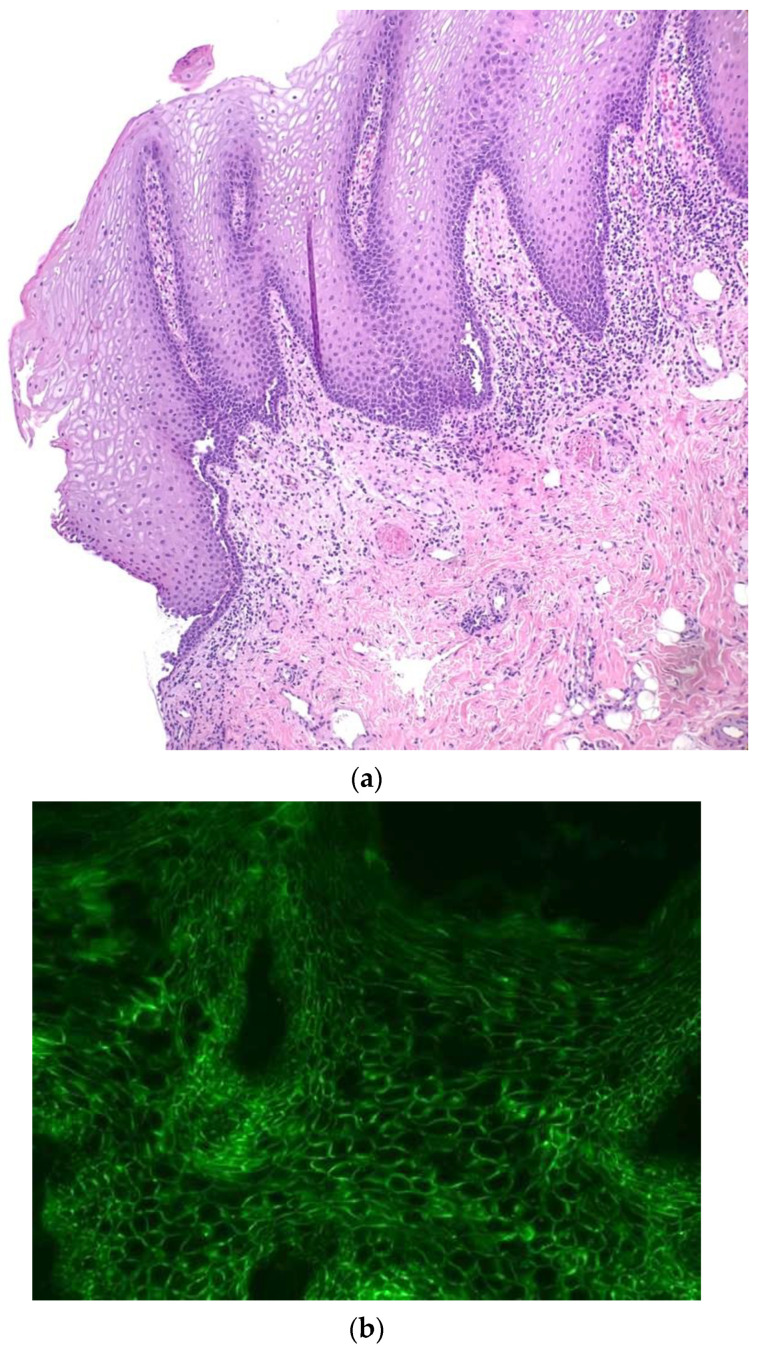
Histology of PV showing intraepidermal suprabasal acantholysis (hematoxylin–eosin staining, 10×); courtesy of Dr. Roberta Manuguerra (**a**). Fluorescent deposits of IgG autoantibodies between intercellular spaces of mucosal keratinocytes (green) in direct immunofluorescence (DIF) (**b**).

The child was hospitalized, and tests were performed including blood chemistry, serology for viruses, gingival swab for herpes simplex and autoimmunity (e.g., ANA reflex, ASCA, ANCA, rheumatoid factor, C3 and C4) tests as well as instrumental tests such as electrocardiography, echocardiography, chest X-ray and ultrasound of the abdomen with normal results except for a mild increase in the inflammatory marker (erythrocyte sedimentation rate (ESR) 37 mm/h). A multidisciplinary approach was undertaken including dermatological, ophthalmological and odontostomatological consultations. Due to suspicion of chronic bullous disease, a mucosal biopsy (Figure 1a), direct immunofluorescence (DIF) (Figure 1b) and a serum anti-epithelial Ig assay and an enzyme-linked immunosorbent assay (ELISA) were performed.

Following the positive result for IgG antibodies BP 180 (74.1 RU/mL; n.v. < 20 RU/mL) and anti-Dsg3 IgG autoantibodies (105.6 RU/mL; n.v. < 20 RU/mlL) and negative result for anti-Dsg1 IgG autoantibodies and anti-epithelium antibodies (1:40), the diagnosis of PV was made (Table 1).

Furthermore, a biochip test was performed, showing positivity for anti-epithelium and anti-spiny cell desmosome antibodies. Nevertheless, the major four criteria for a PV diagnosis, including the coexistence of bullous pemphigoid and PV antibodies, were satisfied [5].

During hospitalization, IV hydration and IV acyclovir (30 mg/kg/day) were administered. Moreover, after dermatological and ophthalmological consultation, steroid therapy with a fully oral dose of prednisone 75 mg/day (1 mg/kg/day for a weight of 75 kilos, weight > 97th percentile for age) was started along with betamethasone and chloramphenicol ocular drops. A few days later, the boy resumed feeding with soft food. The patient was discharged home with steroid therapy until the next dermatological evaluation.

**Figure 2:**
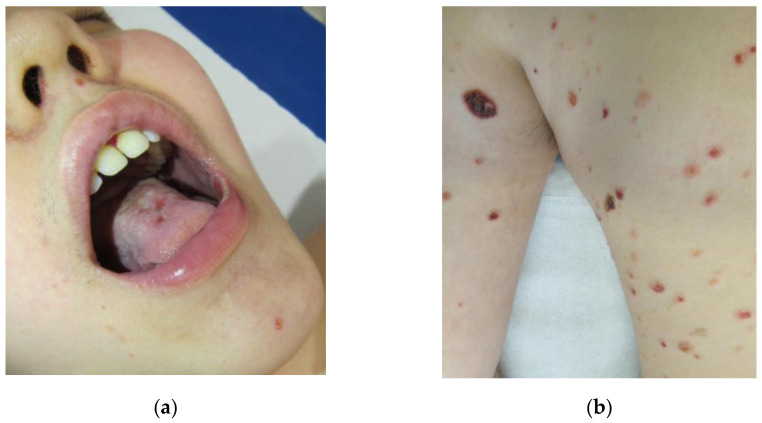
Multiple erosions and widespread de-epithelialization of the oral cavity, particularly of gingival mucosa and hard palate (**a**), and widespread broken vesicles and bullae with crusts of the skin (**b**) in a 12-year-old child with pemphigus vulgaris during second-line therapy.

After one month of follow-up, azathioprine 50 mg/day was added to prednisone 12.5 mg/day to obtain a steroid-sparing effect. After 6 months of immunosuppressive therapy along with supportive therapy in the form of regular nystatin prophylaxis, no improvement was shown in the oral cavity (Figure 2a), and the appearance of skin lesions occurred (Figure 2b). The oral cavity and skin lesions appeared exactly as seen at first admission to the Emergency Unit.

**Figure 3:**
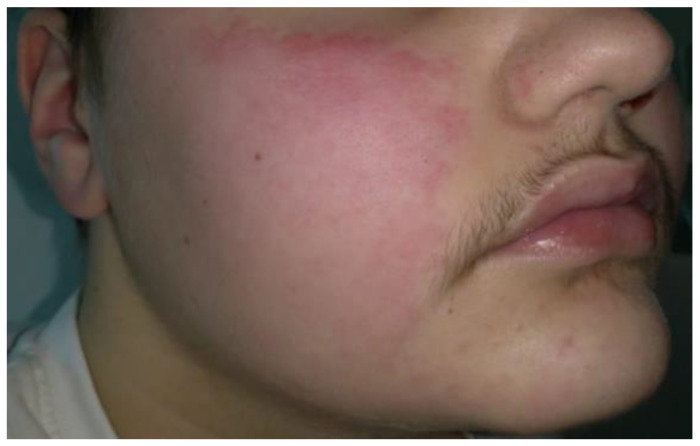
Erythema and urticarial aspects of the cheeks occurred about 30 min after beginning of the rituximab infusion in a 12-year-old child with pemphigus vulgaris.

The continuous relapsing and worsening trend of the disease required the use of R despite the poor data on its use in childhood. The boy was treated with IV R 1 g after IV premedication with methylprednisolone 60 mg + chlorpheniramine maleate 10 mg + paracetamol 1 g. The infusion had to be stopped after about 30 min due to the appearance of an urticarial reaction of the face and widespread erythema of the body with itching and referred difficulties in breathing (Figure 3). Cardiac rate and oxygen saturation level were normal with a minimal decrease in blood pressure (95/65 mm/Hg).

**Figure 4:**
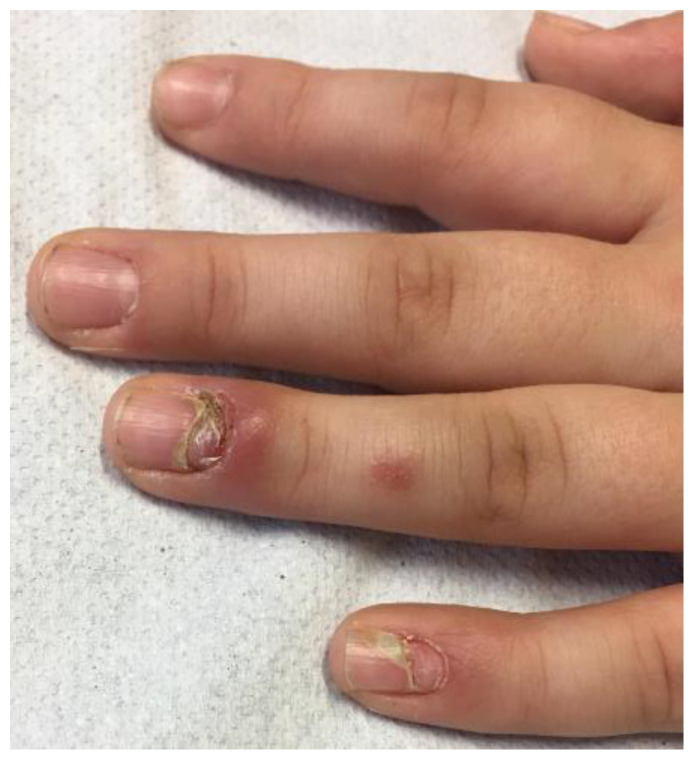
Nail involvement in a 12-year-old child with pemphigus vulgaris after therapy with immunoglobulins and plasmapheresis.

Following this event, prednisone 30 mg/day for 4 weeks with slow scaling was re-started, but the appearance of adverse effects deriving from chronic steroid intake, such as facies lunaris, striae rubrae, buffalo hump, gynecomastia and weight gain, required a reduction in the systemic dosage to 15 mg on alternate days and the evaluation of another treatment. Therefore, IV therapy with immunoglobulins (a total dose of 2 g/kg over 5 days) and seven courses of plasmapheresis once a week with 3 L of plasma with 5% albumin were attempted. Although these last treatments were well tolerated by the patient, with a reduction in antibody anti-DSG1 levels to 57.5 UR/mL, they showed only partial and temporary clinical benefit with, after only a few weeks, further extension of skin lesions, accompanied by subungual involvement, a marker of advanced pathology (Figure 4).

To exclude paraneoplastic PV, also considering the positivity of autoantibodies BP-180, a lymph node station ultrasound and thoraco-abdominal computed tomography were performed, both of which were negative. As a last chance, methotrexate 20 mg/week plus folic acid 5 mg were added to prednisone 25 mg every 2 days for 6 months without a clinical satisfying result. New skin bullae and oral erosions were also present. For this reason, desensitizing therapy to R was considered.

The desensitization was carried out at the hospital. A skin test with R was negative. The desensitization procedure was supervised by personnel (nurses, pediatricians, allergologists, anesthetists) trained to treat severe hypersensitivity reactions. Rescue medications were readily available. Upon arrival of the fasting patient, two peripheral venous accesses were taken. Four desensitizations to R were performed. Premedications were administered one hour before starting each R desensitization. Chlorphenamine, methylprednisolone, paracetamol and omeprazole were administered IV. A 12-step desensitization protocol was planned. The first bag was prepared with an R concentration of 0.034 mg/mL, which was administered IV in the first hour at an increasing infusion rate. During the second hour, the bag with an R concentration of 0.340 mg/mL was administered at an increasing infusion rate. The protocol required that the infusion of the third bag with an R concentration of 3.377 mg/mL be carried out for the following 3 h and 50 min with an increasing infusion rate. However, during the administration of the third bag of R at an infusion rate of 40 mL/h, the patient presented facial erythema along with heat sensation and dyspnea. The infusion was interrupted. Nebulized salbutamol and beclomethasone as well as IV methylprednisolone were administered with rapid resolution of symptoms. A total of 65.798 mg of R was given. PV improved following R administration, but it relapsed after some weeks. So, one month later, the child underwent a second R desensitization using a different protocol. Three bags with drug concentrations of 0.02 mg/mL, 0.20 mg/mL and 2.0 mg/mL were consecutively infused at increasing infusion rates. Five hours after the third bag was started at an infusion rate of 20 mg/mL, the patient presented facial erythema along with heat sensation. A total of 208.88 mg of R was infused. Symptoms resolved after interruption of infusion and administration of IV chlorpheniramine and methylprednisolone. The next day, desensitization continued. After the first two bags, the infusion rate of the third bag was modified. R at the concentration of 2.0 mg/mL was infused at 10 mL/h for 15 min, and then at 15 mL/h for 4.5 h (Table 2). A total of 143 mg of R was infused. The following day, the same protocol was safely used. An R dose of 500 mg was infused in the next three days.

**Figure 5:**
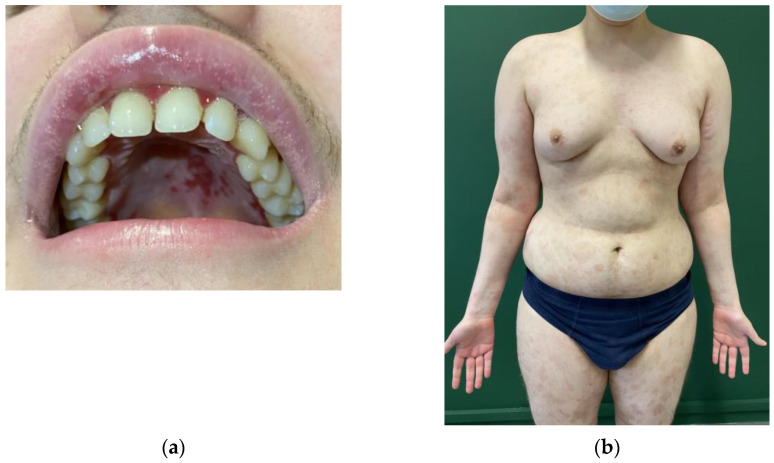
Involvement of the hard palate and gums after 10 days (**a**) and hyperpigmented lesions of the skin and gynecomastia secondary to long-term steroid treatment after 6 months (**b**) in a 12-year-old child with pemphigus vulgaris treated with rituximab after desensitization performed because of a previous hypersensitivity reaction to this drug.

Even if the infusion of R was not at full dose, it allowed for a strong improvement in the disease in about 10 days with complete healing of cutaneous components and residual oral involvement, specifically of the hard palate and the gums (Figure 5a). After 6 months, general outcomes consisted of diffuse hyperpigmented lesions, with residual gynecomastia and striae distensae. No new blisters or itching were reported (Figure 5b).

At 6 months, the oral mucosa was ameliorated, with a slight involvement of the inferior gum persisting without any symptoms. Furthermore, the therapy led to an important improvement in quality of life, considering even the psychological impact on a patient of such a critical age.

## 3. Discussion

PV is a life-threatening autoimmune bullous disease that generally affects adults between the fifth and seventh decades of life. It is extremely rare, and the incidence in the pediatric population is 1–4% with no predominance of gender [6]. The pathogenesis includes the formation of pathogenic IgG autoantibodies directed against structural proteins of desmosomes, special structures responsible for interkeratinocytes’ adhesion. In particular, Dsg1 and 3 are transmembrane glycoproteins of mucosal and cutaneous districts, respectively. The attack of these proteins leads to an intraepidermal acantholysis and intercellular deposition of IgG and C3 in the tissue, with the consequent formation of intraepidermal blisters. The formation of anti-Dsg autoantibodies is usually related with the activity of PV. [5]. Clinically, persistent mucosal involvement is generally the first sign, with the oral cavity being the major site of disease, involving the gingiva, soft and hard palate, floor of the mouth, posterior pharynx labial mucosa and less frequently the tongue, with erosions and severe inflammation. Pain and dysphagia are usually present, making it difficult to eat. Other mucosae can be affected, such as the nasal, conjunctival, esophagus, pharyngo-laryngeal mucosa and genital or anal mucosa. Skin lesions can occur also after several months of mucosal involvement, as in our case [7].

The severity of the disease is due to skin and mucosal detachment that causes fluid and protein loss, exposing the patient to possible superinfections, the major cause of mortality [8]. Pediatric patients with PV have a 2.4-fold increase in mortality compared with the general population, although its formerly relatively high mortality (ranging from 5% to 30%) is lower nowadays thanks to therapeutic advancements [9].

The diagnosis of PV is based on four major criteria: clinical presentation, histopathology, DIF of a perilesional skin or mucosal biopsy and serological detection of autoantibodies against epithelial cell surface using indirect immunofluorescence (IIF) and/or enzyme-linked immunosorbent assay (ELISA Dsg1 and Dsg3) [5].

The diagnosis of PV is not always simple, and differential diagnosis should include, even in children, other diseases characterized by dermo-epidermal detachment, such as bullous impetigo, staphylococcal scalded skin syndrome and Steven–Johnson syndrome/toxic epidermal necrolysis [10]. Additionally, in our case, at the time of the first admission to our hospital, the presence of persistent vesicles and erosions in the oral cavity primarily oriented the diagnosis towards a more common pediatric infectious disease (i.e., herpangina, HSV or stomatitis).

Low-dose oral corticosteroids represent a mild form of therapy. In moderate-to-severe forms, the prednisone dosage must be increased to 1–1.5 mg/kg/day and possibly integrated with other immunosuppressive drugs (such as azathioprine and/or mycophenolate mofetil). R, a chimeric monoclonal antibody against CD20, represents today a first-line therapy in adulthood, with a therapeutic scheme of two infusions of 1 g two weeks apart with or without oral prednisone [2,3,5].

According to the literature, R appears to be well tolerated in children with PV, with the commonest adverse events being bacterial and viral infections and the rarest complications being B-cell depletion and hypogammaglobulinemia [3,11]. A recent systematic review of five pediatric cases did not show any adverse events associated with R and concluded that it could be better tolerated than alternative immunosuppressive regimens [12]. R must especially be considered in refractory diseases or contraindications of corticosteroids [6]. It is well known that in a subset of patients receiving R for the first time, early-infusion-associated adverse reactions (EIARs) may occur, including a combination of fever, chills, nausea, vomiting, hypotension, bronchospasm, rhinitis, angioedema, urticaria and/or flushing. These reactions are more frequent in patients treated for lymphoma and patients with multiple sclerosis who have not received high-dose corticosteroid pretreatment [13].

In our case, we observed failure along with other immunosuppressive drugs, immunoglobulins and plasmapheresis. In comparison with a recent case treated with four cycles of plasmapheresis, during which 90% of the erosions healed, our patient underwent seven sessions without any resolution and even experienced skin and mucosal worsening [14].

The management of our clinical case involved the use of all available treatments for PV, with the difficulty of adapting adult guidelines to the pediatric patient according to their needs. The first obstacle was the use of corticosteroids that were poorly tolerated by the child, who manifested significant adverse effects that also affected their psychological sphere. Moreover, nowadays we cannot exclude in our case a role of obesity in both pathogenesis and treatment failure. To date, there are only two reports in the literature showing a possible association between pemphigus and metabolic syndrome and its components. Further studies will be necessary to find a strong correlation between overweight/obesity and PV.

To the best of our knowledge, this is the first report of a child with severe PV resistant to conventional therapies and with a hypersensitivity reaction to R. The desensitization test for R made it possible to find the right combination of infusion rate and dosage, leading to the remission of the clinical picture and an improvement in the patient’s quality of life, which was previously severely compromised even in basic activities of daily living.

Despite the high cost of the molecule, its superior efficacy and durability may ultimately lead to lower overall system costs owing to more rapid recovery and fewer relapses.

## 4. Conclusions

This case highlights that, despite PV being extremely rare in pediatric populations, this diagnosis should not be discounted in cases of unresponsive gingivostomatitis-like lesions, integrating clinical manifestations with laboratory findings. An early diagnosis can play a decisive role in treatment strategies and improving a patient’s quality of life. In cases of severe clinical manifestations with failure to first- and second-line therapies, R represents a valid option in pediatric ages, and desensitization tests should be recommended in the presence of allergy or hypersensitivity to this drug. A multidisciplinary approach is moreover mandatory to obtain better results for the patient. Further studies will be necessary to define treatment guidelines for children and adolescents, allowing for appropriate management of PV in pediatric ages.

## Figures and Tables

**Table 1:** Autoantibodies in a 12-year-old child with pemphigus vulgaris.

Autoantibodies	Admission	After 20 Months	After 22 Months	After 32 Months
Anti-epithelium	Positive (1:40)	Positive (1:80)	Positive (1:80)	Positive (1:80)
Ab anti-BP-180 IgG (ELISA)	74.1 UR/mL (positive > 20)	35.5 UR/mL (positive > 20)	5.1 UR/mL (positive > 20)	-
Ab anti-desmoglein 1 IgG (ELISA)	16.2 UR/mL (positive > 20)	>200 UR/mL (positive > 20)	57.5 UR/mL (positive > 20)	57.8 UR/mL (positive > 20)
Ab anti-desmoglein 3 IgG (ELISA)	105.6 UR/mL (positive > 20)	>200 UR/mL (positive > 20)	154.5 UR/mL (positive > 20)	231.1 UR/mL (positive > 20)

ELISA, enzyme-linked immunosorbent assay.

**Table 2:** Modified protocol used for desensitization to rituximab in a 12-year-old boy with pemphigus vulgaris who showed a previous anaphylactic reaction to rituximab: solutions’ concentrations (a) and rates (b).

**(a)**								
		**Volume (mL)**			**Concentration (mg/mL)**		**Amount of Drug in Each Solution (mg)**	
Solution 1		250			0.02		0.011	
Solution 2		250			0.20		0.114	
Solution 3		250			2.00		1.131	
**(b)**								
**Step**	**Solution** **(n)**		**Rate** **(mL/h)**	**Time** **(min)**		**Administered Drug** **(mg)**		**Cumulative Dose** **(mg)**
1	1		2	15		0.01		0.01
2	1		4	15		0.02		0.03
3	1		10	15		0.05		0.08
4	1		20	15		0.1		0.18
5	2		4	15		0.2		0.38
6	2		10	15		0.5		0.88
7	2		20	15		1		1.88
8	2		40	15		2		3.88
9	3		10	15		5		8.88
10	3		15	270		135		143.88

## Data Availability

All the data are included in the manuscript.
